# Analytical and Numerical Modeling of Stress Field and Fracture in Aluminum/Epoxy Interface Subjected to Laser Shock Wave: Application to Paint Stripping

**DOI:** 10.3390/ma15103423

**Published:** 2022-05-10

**Authors:** Kosmas Papadopoulos, Konstantinos Tserpes

**Affiliations:** Laboratory of Technology & Strength of Materials, Department of Mechanical Engineering & Aeronautics, University of Patras, 26500 Patras, Greece; kosmaspapadopoulos@upnet.gr

**Keywords:** laser shock, paint stripping, shock wave propagation, spall fracture, finite element analysis

## Abstract

In this paper, analytical and numerical models have been developed to compute the stress field and predict fracture of the aluminum/epoxy interface subjected to laser shock loading, in the frame of the investigation of the paint stripping process. An explicit finite element (FE) model combined with the cohesive zone modeling (CZM) method, an analytical stress analysis model, and a spall fracture model have been developed. The numerical model has been calibrated and validated against tests in terms of the stripping pattern, while the analytical models have been compared with the numerical model. The models were combined to generate computational tools for decreasing computational effort. The FE model with the CZM is the most accurate tool although it is the most computationally expensive. The spall fracture model gives trusted estimations of the spall strength of the interface which are very sensitive to the interface thickness and when incorporated into a continuum FE-based damage model can predict the stripping initiation faster than the FE model with the CZM. The analytical stress analysis model can be used to efficiently describe the shock wave propagation into the material system, but it can give only a rough estimation of the tensile stress at the epoxy, which when combined with the spall strength does not give reliable predictions of the stripping initiation. The three models require as input different material properties, some of which are very difficult to determine. Nevertheless, the availability of accurate material parameters and properties of the aluminum, the epoxy, and, especially, their interface can significantly improve the efficiency of the developed models.

## 1. Introduction

Laser shock applications in materials processing have grown significantly in recent years. For instance, laser shock peening (LSP) is used to create a residual compressive field on the material’s surface to enhance the fatigue life [1,2,3] while this residual stress field also improves the surface’s mechanical and corrosion resistance [4]. The laser shock adhesion test (LASAT) employs tensile stresses, resulting from the release wave, to break defect-related weak bonds in the adhesive bondline without damaging the substrates [5,6]. Different approaches to the laser shock other than the mono pulse impact have been also applied. The symmetrical laser shock has been used for creating controlled delamination in composites [7] and for the weak bond inspection [8]. The laser-delayed double shock has been attempted as an attractive method for improving the LASAT [6,9]. Moreover, similarly to the LASAT method, the laser shock is also capable of disassembling bonded parts without damaging the substrates [10].

In addition to the above applications, the laser shock can be used for paint stripping of aeronautical and aerospace structures [11,12] which fundamentally differs from laser paint stripping in the use of thermal effects for ablation of paint [13,14]. In conventional paint stripping, hazardous chemicals and media blasting are used, thus damaging the substrate. This brings the need for the development of more sustainable methods, with less environmental impact. A candidate method is laser shock paint stripping (LSPS). In LSPS, the objective is the removal of the paint without causing any damage to the substrates, which might counterbalance the recycling and reusing processes. The stripping can be expressed as the fracture of the interface between the paint and the substrates. This fracture because of the dynamic nature of the laser shock loading and the high strain rates that take place is examined through the *spall fracture mechanism*.

To optimize the LSPS method, a good understanding of the physical phenomena that take place is needed. Aside from the numerous experimental works, several analytical [15,16,17,18,19,20,21,22] and numerical [1,2,5,6,10,23] models have been developed to describe the shock wave propagation and its interaction inside a solid material. This is not an easy task due to the nature of the shock wave, which acts as a discontinuity inside the material. Analytical models describe mathematically the shock wave propagation through the *jump equations* which arise from the conservation of mass, the balance of momentum, and energy principles, solving thus, the discontinuity problem of the shock loading. Fracture mechanics, and especially the spall mechanism [20,24,25,26], have contributed by describing the failure of materials under high strain rate conditions. In addition, many works have addressed the magnetohydrodynamics (MHD) of plasma matter interaction. HELIOS numerical code has been used to simulate the plasma expansion between the confinement layer and material [27]. Ablation pressure prediction has been achieved using the ESTHER code by [28,29]. Numerical models such as the finite element method (FEM) might prove very useful for the study of the shock wave propagation inside a solid material [30,31] as they can provide detailed stress fields in shape and values at different times as the shock wave propagates. A main disadvantage of the FE method is the large computational effort it requires, which depends both on the characteristics of the model and the available computational resources. On the other hand, an analytical solution is obtained at a fraction of the time of a FE simulation, still, with less accuracy. Moreover, analytical models even though they do not have the capacity to provide a detailed description of the stress field, could be used for a preliminary estimation of stresses and the first assessment of fracture.

The stripping condition can be either treated as a failure of the paint or as a fracture of the interface. Interface mechanics constitute one of the most complex areas of mechanics because of the uncertainty they contain. A bond can be characterized by four different adhesion theories, the mechanical, the absorption, the electrostatic, and the diffusion theory [32]. Thus, it is difficult to distinguish the percentage of the influence of each one on the bond strength. Numerical models aim to simulate the interface via two approaches, the cohesive zone modeling (CZM) method [33,34] and the virtual crack closure technique (VCCT) [35] based on the linear elastic fracture mechanics. Provided the necessary input parameters are available, both approaches give very accurate and useful results. In addition, phase-field models have proven a useful tool for fracture mechanics and have been used widely for composite materials’ failures [36,37] and to predict interface debonding both without the CZM method [38] and in combination [39].

When high strain rate phenomena take place, the fracture begins as a spall fracture in the microstructure of the material [20,24]. The theory behind the spall fracture is based on the energy balance approach, in which when exceeded, the cohesive energy spall initiates. Additionally, there are two types of spall fracture, the brittle and the ductile, which depend on the load and material’s characteristics. The material’s quasi-static strengths are surpassed because of the high magnitude of the developed stress fields, especially the tensile, and new strength values emanating from the dynamic spall theory must replace the static ones. Combining the dynamic nature of the shock wave with the theory of the spall fracture, one can obtain a framework to study how the shock wave causes fracture within a material or at the interface between two materials analytically. Of course, several assumptions and simplifications must be taken into account to define the problem and overcome any barriers.

In the present paper, we have developed computational tools for decreasing computational effort by combining numerical and analytical models to study the mechanics of the laser shock stripping, compute the stress field at the aluminum/interface, and predict the stripping initiation in an aluminum/epoxy specimen. Experimental results from previous works have been for calibrating, where necessary, and validating the models. The paper is structured as follows. Section 2 describes the specimen, the loading, and the shock wave propagation inside the material system. In Section 3, the experimental setup used in [11] to conduct the LSPS tests is briefly presented. Section 4 describes the FE model including the CZM module, Section 5 the analytical stress analysis model, and Section 6 the spall fracture model. In Section 7, the results of analyses conducted by the different computational tools are described. Section 8 summarizes the main findings and presents the conclusions of the work.

## 2. Problem Statement and Approach

**Figure 1:**
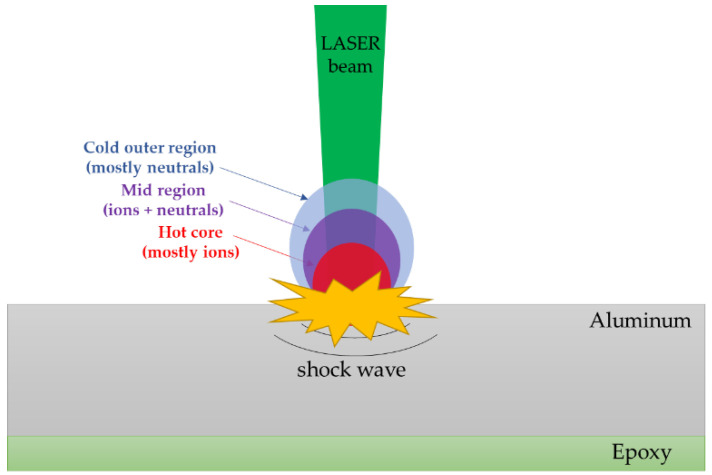
Schematic of the laser shock wave generation and the specimen’s configuration.

Consider an aluminum plate covered with a layer of epoxy paint [11,12] (Figure 1). The laser is applied on the aluminum side. When the laser–matter interaction takes place, *plasma* is generated through evaporation, ionization, and expansion [12]. The region of plasma close to the surface is called *core* and it is the hottest and densest part. The material in this region is mostly in an ionized state. The second region is called *mid-region* and there are both ions and neutrals. Last, the outer part is called the *cold outer region* and consists of mostly neutrals [40]. The expansion of plasma leads to the creation of a shock wave that propagates inside the material. A schematic representation of the process and the different areas can be seen in Figure 1.

**Figure 2:**
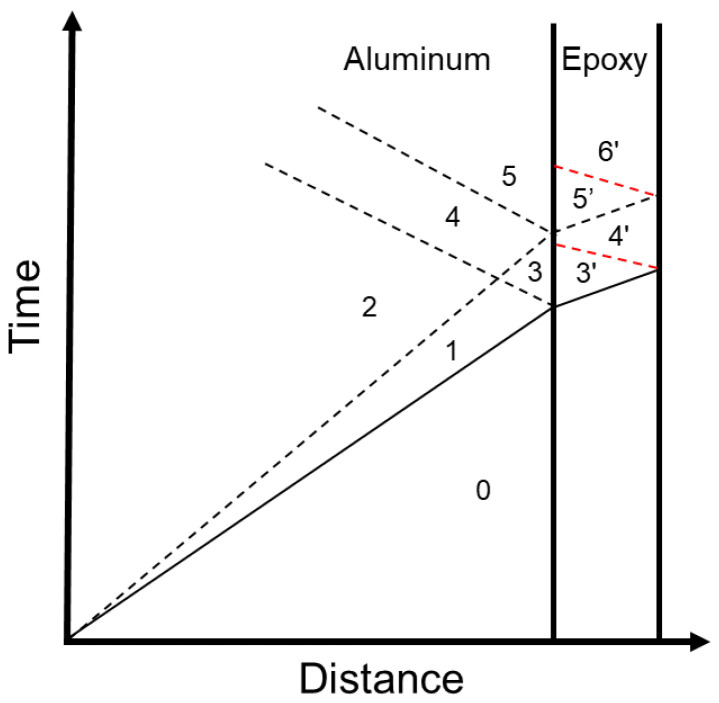
Schematic representation of the shock wave propagation into the aluminum/epoxy material.

In the following, the different interactions of the propagating shock inside the specimen are described. Those interactions are schematically presented in the diagram in Figure 2. The solid line indicates the elastic precursor, the dashed line the plastic shock, and the red line the decompression shock.

- First, the loading is applied to the aluminum free surface by the laser–plasma and a shock wave initiates inside the material;
- The shock wave is separated into an *elastic precursor* that takes the material from state 0 to state 1 and to a *plastic shock* that takes the material to state 2;
- When the loading is removed from the surface of the aluminum a *decompression elastic-plastic shock* initiates;
- The elastic part of the compressive shock interacts with the aluminum/epoxy interface and a left propagating compressive shock moves inside the aluminum taking to it to state 3;
- This left propagating shock interacts with the plastic shock and takes the material from state 3 to state 4;
- Part of the shock wave starts propagating inside the epoxy material taking it to state 3′;
- When the shock wave that is propagating inside epoxy meets the free surface it reflects a left propagating decompression shock taking epoxy to state 4′;
- The plastic shock then reflects from the interface and takes the aluminum from state 4 to state 5, while part of it propagates inside the epoxy, taking it to state 5′ and reflects from the free surface to a decompression shock that takes the epoxy to state 6′.

Using this generated shock wave the paint removal is achieved through the propagation of states 4′ and 6′ to the interface. The stripping can be distinguished into two main domains: the failure of the paint and the fracture of the interface between the paint and the substrate. It is worth mentioning that in the present work the stripping by the laser shock is examined as a purely mechanical process without considering any thermal effects. The study is focused on how the shock wave initiates fracture and spall fracture of the interface and in some cases how this fracture propagates with time. This will be realized via analytical and numerical models, while experiments conducted in [11] will be used as a basis for the validation and calibration of the models.

**Figure 3:**
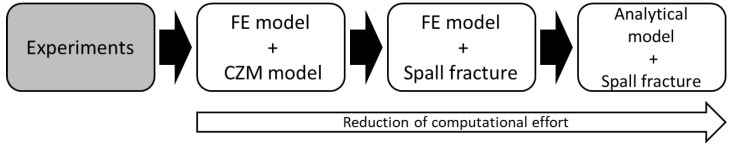
The approach of the paper.

The objective of the present work is twofold: to study the mechanics of the laser shock stripping by tests, analytical and numerical modeling, and to develop predictive tools for stripping initiation. The development of predictive tools is a stepwise process moving towards the reduction of the required computational effort. The starting point is the tests, and each step is validated by a previous step. Figure 3 gives a schematic illustration of the approach. After the tests, a FE model combined with the CZM method [12] is developed to compute the evolution of stress with time within the materials and to simulate stripping initiation and propagation. The FE model has been validated against tests in terms of VISAR (Velocity Interferometer System for Any Reflector) measurements in [12]. Next, the stresses computed by the FE model are compared against the spall strength of the aluminum/interface, which is derived from the *dynamic spall strength theory*, to predict the striping initiation. In parallel, an analytical model capable of computing the stresses at the interface is developed and validated against the FE model. Finally, the analytical model is combined with the spall strength to achieve the fastest prediction of the stripping initiation for a given laser intensity. Apart from stresses, stripping initiation predictions are validated against experimental observations.

## 3. Experimental Setup

**Figure 4:**
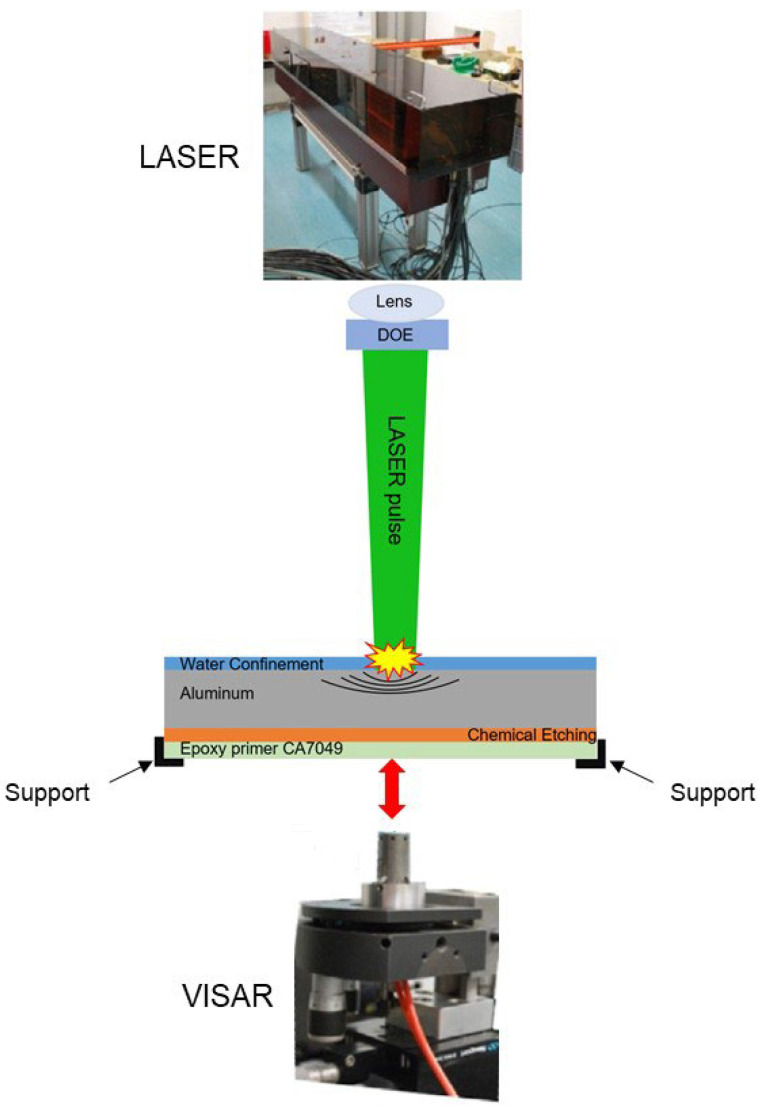
The experimental set-up of the laser shock (PIMM, Paris, France).

The experimental setup is hosted by PIMM Laboratory in Paris, France. It consists of a Gaia HP laser from THALES company (Paris, France). This is an Nd:YAG laser with a 7.2 ns pulse duration, 14 J of energy (Gaussian pulse), and a 2 Hz of repetition rate at 532 nm wavelength. A single beam of 1.75 GW/cm^2^ intensity was used, and energy calibration was done via a calorimeter (QE50LP-H-MB-QED, Gentec, Quebec, QC, Canada). For the conducted experiments, the spot size was kept constant at 4 mm through a lens with a focal length of 198 mm. In addition, the diffractive optical element (DOE) was used to obtain a top-hat-shaped beam with an equal beam distribution along the spot size [8,12]. The materials under examination were the aluminum AA 2024-T3 with a chemical etching surface treatment and the epoxy primer CA7049 produced by RESCOLL, Pessac, France [12]. The material’s response was assessed by measuring the doppler shift of mono mode probe laser (532 nm) that is reflected on the back face of the accelerated target via shock wave [11,41]. A schematic description of the experimental setup is shown in Figure 4.

**Figure 5:**
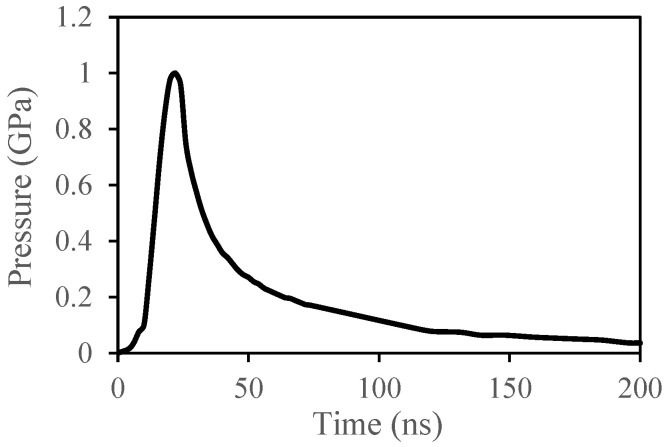
The pressure profile.

By applying the plasma confinement, the ablation of the plasma pressure is achieved. In the present installation, a water confinement [42] was used and a duration of two times longer and a magnitude of four times higher was realized [43,44]. In addition, the water confinement was used for the absorption of thermal effects of the laser–plasma–matter interaction. Figure 5 represents the adjusted pressure temporal profile produced by laser–plasma–matter interaction [1,44]. This pressure profile represents the loading that has been applied to the models.

## 4. Numerical Model

**Figure 6:**
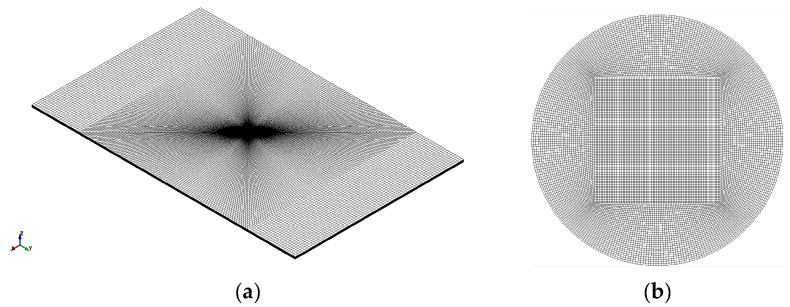
(**a**) FE mesh of the specimen, (**b**) FE mesh of the circular spot.

To compute shock wave stresses and to simulate the stripping, a 3D FE model was developed in [11,12] using the explicit FE software LS-DYNA. The modeled specimen consists of an aluminum substrate with a thickness of 1 mm and an epoxy thickness of 0.025 mm. The modeled laser spot’s diameter was 4 mm. The boundary conditions that have been applied to the model restrict the normal displacement (along the *z* axis) at the bottom left and the right edge of the epoxy. To prevent any non-physical modes, the hourglass energy was computed with the *CONTROL_ENERGY keyword and restricted to be less than 5% of the total energy with the *CONTROL_HOURGLASS. A mapped FE mesh consisting of different areas was developed (Figure 6). For the laser spot, represented by a circular area in the middle specimen, a fine mesh was developed using elements with a size of 0.027 mm (Figure 6b), whereas for the rest of the specimen a coarser mesh with a size of 0.042 mm was adopted. The mesh size through the thickness was kept constant at 0.005 mm. Thus, a denser mesh was created under the loading area and especially through the z axis which is the wave’s propagation axis. The element type that was used for both materials is a 3D 8-noded solid with one integration point (ELFORM = 1).

Simulation of the aluminum’s behavior was performed using the *MAT_15_JOHNSON_COOK material model while for the epoxy the *MAT_10_ELASTIC_PLASTIC_HYDRO material model was used. For both materials, the Gruneisen equation of state [12] was implemented. The mechanical properties used in the material models are listed in Table 1.

The stripping was simulated using the CZM method. Zero thickness 8-noded, 4-point cohesive elements (ELFORM = 19) cohesive elements were used between the aluminum and the epoxy. CZM elements share the same nodes with the aluminum and epoxy elements. The cohesive behavior was simulated using a bi-linear mixed-mode I + II traction-separation law [33]. The input parameters are the critical energy release rates of the interface under mode I ($G_{IC}$) and II ($G_{IIC}$) fracture modes, the peak normal and shear stresses, and the interface’s density. These fracture properties can be obtained by mode I and II experiments. However, for the specific material system, it is very difficult to manufacture double cantilever beam (DCB) and end-notched flexure (ENF) specimens. Therefore, a calibration of the traction-separation law against tests was performed. As the stripping is a mode I dominated phenomenon [12], thus the calibration process involved the variation of $G_{IC}$ within the range of 0.95–1.02 mJ/mm^2^.

## 5. Analytical Modeling of Stress Field

A shock wave is a propagating surface in which the displacement is continuous but the mass density, the particle velocity, the stress, and other field variables are discontinuous. For the characterization of plane shocks embedded in smooth uniaxial motion, the following jump conditions were developed [18]:

(1) $$\mathrm{Conservation}\;\mathrm{of}\;\mathrm{mass}:\;\rho_{R}U_{s}〚-v〛=〚\dot{x}〛$$

(2) $$\mathrm{Balance}\;\mathrm{of}\;\mathrm{momentum}:\;\rho_{R}U_{s}〚\dot{x}〛=〚-t_{11}〛$$

(3) $$\mathrm{Balance}\;\mathrm{of}\;\mathrm{energy}:\;\rho_{R}U_{s}〚\varepsilon +\frac{1}{2}{\dot{x}}^{2}〛=〚-t_{11}\dot{x}〛$$

where ρ is the material’s density, $U_{s}$ is the shock velocity, $\dot{x}$ is the material’s velocity, $t_{11}$ is the Cauchy stress, v is the specific volume and ε is the specific internal energy. Some further assumptions and considerations must be made before reaching the analytical solution. The medium is considered a homogenous and isotropic elastic-plastic material that is subjected to small uniaxial deformation. Additionally, the analysis is restricted to weak shocks so the thermal variables can be neglected.

The compression shock waves are produced by a sudden application of a uniform compressive load. While the shock has a compressive amplitude lower than the *Hugoniot Elastic Limit* (*HEL*), it involves only the elastic response. A shock of greater amplitude than the *HEL* is unstable, and it separates into two shocks: the elastic precursor that propagates at the elastic wave speed $C_{0}$ and the plastic shock that propagates at the bulk wave speed $C_{B}$, given by the following equations:

(4) $$\mathrm{Elastic}\;\mathrm{wave}\;\mathrm{speed}\;C_{0}=\sqrt{\frac{\lambda_{R}+2\mu_{R}}{\rho_{R}}}$$

(5) Bulk wave speed CB=λR+23μRρR

where $\lambda_{R}=\frac{E\nu }{\left(1+\nu )(1-2\nu \right)}$ and $\mu_{R}=G$ are the first and second Lame parameters, respectively, where E is Young’s modulus, ν is Poisson’s ratio, and G is the shear modulus.

Meanwhile, decompression shocks are produced inside the material by the sudden removal of the applied compressive load or by the interaction of a compression wave with a material’s boundary. For the analysis of a decompression wave, three different ranges must be considered. The first range includes only the linear elastic response, while $-t_{11}\leq t_{11}^{HEL}$. In the second range, $t_{11}^{HEL}<-t_{11}\leq 2t_{11}^{HEL}$, the decompression to a zero-stress state is done by a single elastic decompression shock. For stresses $2t_{11}^{HEL}<-t_{11}$, the decompression shock is unstable and separates into an elastic and a plastic shock.

Last, it is important to mention the two types of reflection surfaces and how the shock interacts with them. The reflection from an unrestrained boundary acts as an interface with an incompressible body and the shock that is reflected is compressive. The shock that is reflected from an unrestrained boundary is a decompression wave.

The correlation between the laser’s intensity and the applied pressure to the material has been extensively studied in [44,45,46]. When a high-power laser beam is applied to a material it leads to the plasma generation of high temperature which initiates a shock wave inside the material. Equation (6) describes the relationship between the maximum applied pressure, material properties, and laser parameters.

(6) $$P_{max}=0.01\sqrt{\frac{\alpha }{2\alpha +3}}\sqrt{ZI_{0}}$$

(7) $$\frac{2}{Z}=\frac{1}{Z_{1}}+\frac{1}{Z_{2}}$$

(8) $$Z_{i}=\rho_{i}D_{i}\;(i=1,2)$$

(9) $$D_{i}=C_{0}+Su\;(i=1,2)$$

where $I_{0}$ (GW/cm^3^) is the laser’s intensity, α is the part of the energy being used for the gas ionization, Z (g cm^−2^/s^−1^) is the material’s acoustic impedance, $C_{0}$ is the sound speed inside the material, u is the material’s velocity, S is a dimensionless coefficient and i is the indication factor of different materials.

The calculation of *HEL* values is of great importance because it describes state 1 (Figure 2), which is the state behind the elastic precursor. *HEL* values were computed using the following equations:

(10) $$t_{11}^{HEL}=\frac{\lambda_{R}+2\mu_{R}}{2\mu_{R}}Y$$

(11) $${\tilde{E}}_{11}^{\left(1\right)}=-\frac{1}{\rho_{R}C_{0}^{2}}t_{11}^{HEL}\equiv -{\tilde{E}}_{11}^{HEL}$$

(12) $${\dot{x}}^{\left(1\right)}=\frac{1}{\rho_{R}C_{0}}t_{11}^{HEL}\equiv {\dot{x}}^{HEL}$$

where $-{\tilde{E}}_{11}$ is the component of the strain tensor and $\dot{x}$ is the material’s velocity. When the jump conditions are applied to the plastic wave Equations (13) and (14) that describe the state 2 (Figure 2) are derived, given the stress as a boundary condition equals to $-t_{11}^{\left(2\right)}=2.1\;\mathrm{GPa}$.

(13) $${\tilde{E}}_{11}^{\left(2\right)}=\frac{1}{\rho_{R}C_{B}^{2}}\left\{t_{11}^{\left(2\right)}+\left(\frac{C_{0}^{2}-C_{B}^{2}}{C_{0}^{2}}\right)t_{11}^{HEL}\right\}$$

(14) $${\dot{x}}^{\left(2\right)}=\frac{1}{\rho_{R}C_{B}}\left\{-t_{11}^{\left(2\right)}-\left(\frac{C_{0}-C_{B}}{C_{0}}\right)t_{11}^{HEL}\right\}$$

State 3 (Figure 2) is the state behind the reflected plastic compressive shock, and it is characterized by the following equations:

(15) $$t_{11}^{\left(3\right)}=-\frac{C_{0}+C_{B}}{C_{0}}t_{11}^{HEL}$$

(16) $${\tilde{E}}_{11}^{\left(3\right)}=-\frac{C_{0}+C_{B}}{\rho_{R}C_{0}^{2}C_{B}}t_{11}^{HEL}$$

(17) $${\dot{x}}^{\left(3\right)}=0$$

The interaction between the reflected plastic shock and the incident plastic shock produces the state 4 (Figure 2) which is described by:

(18) $$t_{11}^{\left(4\right)}=t_{11}^{\left(2\right)}-\frac{C_{B}}{C_{0}}t_{11}^{HEL}$$

(19) $${\tilde{E}}_{11}^{\left(4\right)}=\frac{1}{\rho_{R}C_{B}^{2}}\left[t_{11}^{\left(2\right)}+\frac{C_{0}^{2}-C_{0}C_{B}-C_{B}^{2}}{C_{0}^{2}}t_{11}^{HEL}\right]$$

(20) $${\dot{x}}^{\left(4\right)}=\frac{1}{\rho_{R}C_{B}}\left[-t_{11}^{\left(2\right)}-t_{11}^{HEL}\right]$$

The final interaction inside the aluminum is the reflection of the plastic shock from the interface to a plastic compression shock that takes material from state to state 5 (Figure 2), described by:

(21) $$t_{11}^{\left(5\right)}=2t_{11}^{\left(2\right)}+\frac{C_{0}-C_{B}}{C_{0}}t_{11}^{HEL}$$

(22) $${\tilde{E}}_{11}^{\left(5\right)}=\frac{1}{\rho_{R}C_{B}^{2}}\left[2t_{11}^{\left(2\right)}+\frac{C_{B}\left(C_{0}-C_{B}\right)}{C_{0}^{2}}t_{11}^{HEL}\right]$$

(23) $${\dot{x}}^{\left(5\right)}=0$$

For the analytical solution, the algebraic equations were implemented into MATLAB and the results will be presented in the form of Hugoniot graphs. All equations that were used are functions of *HEL* values, mass density, and shock wave elastic and plastic speeds.

## 6. Analytical Modeling of Spall Fracture

Spall is the process of internal failure or rupture of condensed media through a mechanism of cavitation due to stress in excess of the tensile strength of the material [20,24]. When a threshold tensile stress is achieved, void nucleation occurs and spall fracture initiates. Dynamic spall is a collective nucleation, growth, and coalescence process that depends on the pre-existing and evolving microstructure of the body. In certain materials, when very rapid tensile loading rates are achieved, theoretical and experimental spall strengths are close [24]. The most common experimental method is the flat impact of plates of material, while the theoretical spall strengths arise from energy balance analysis when the elastic energy equals the cohesive energy.

For a given metal, spall can be a transition from brittle to ductile, with increasing the strain rate. Brittle and ductile spall is defined as a dynamic failure through the activation, growth, and coalescence of a network of cracks for the former and similar failure through the growth and linkup of spherical voids, for the latter [24]. The critical value of the strain rate for spall initiation is derived by:

(24) $${\dot{\varepsilon }}_{crit}=\sqrt{\frac{8}{9}\frac{E^{2}{\left(Y\varepsilon_{c}\right)}^{3}}{\rho K_{C}^{4}}}$$

where Y is the yield stress, $\varepsilon_{c}=0.18$ [24] is the critical void volume fraction and $K_{C}$ is the fracture toughness of the material. It is worth mentioning that the yield stress and the fracture toughness of the material were assumed to be independent of the strain rate.

At relatively gentle strain rates it is suggested that the spall process involves the activation and growth of mature ductile cracks before coalescence and spall failure occurs, which is a fracture toughness-controlled phenomenon. On the contrary, at higher strain rates it is suggested that spall in the same metal is a process of hole growth and coalescence without the formation of mature cracks, which is a flow stress-controlled phenomenon [24]. By an energy balance analysis [20,24] the brittle and ductile spall strengths arise and are described by the following equations

(25) $$\mathrm{Brittle}\;\mathrm{spall}\;\mathrm{strength}:\;P_{s}^{B}={\left(3\rho c_{0}K_{C}^{2}\dot{\varepsilon }\right)}^{1/3}$$

(26) $$\mathrm{Ductile}\;\mathrm{spall}\;\mathrm{strength}:\;P_{s}^{D}={\left(2\rho c_{0}^{2}Y\varepsilon_{c}\right)}^{1/2}$$

If the focus is on the epoxy the brittle spall strength must be chosen, because of the brittle nature of the epoxy material. Equation (25) can be re-written in the following form:

(27) $$Ut\geq \frac{3\gamma }{c_{0}}$$

where $U=P^{2}/2\rho c_{0}^{2}$ is the elastic energy density, $\gamma =K_{C}^{2}/2\rho c_{0}^{2}$ is the fracture energy release rate, and t is the time domain [24]. If we substitute U and γ in Equation (27) and we solve for P, then Equation (28) gives the stress threshold for the initiation of brittle spall:

(28) $$P_{th}\geq \sqrt{\frac{6\rho c_{0}\gamma }{t}}$$

From fracture mechanics $G_{C}$ can be also expressed as the energy per unit area from crack initiation to crack exiting time:

(29) $$G_{C}=\frac{\int_{U_{ci}}^{U_{cf}}FdU}{A}$$

where $U_{ci}$ and $U_{cf}$ are the relative displacements corresponding to crack initiation time and crack exiting time, respectively, [47], and A is the surface area. Equations (28) and (29) will be used for an approximate calculation of the aluminum/epoxy interface stress threshold, where the spall (stripping) initiates.

## 7. Computational Tools and Results

### 7.1. Calibration of the CZM

**Figure 7:**
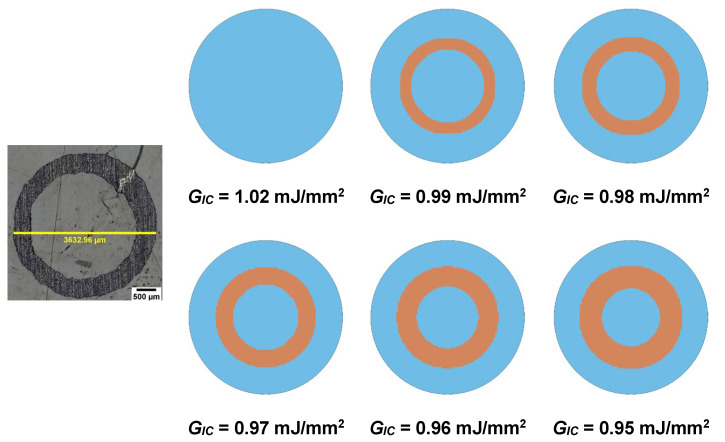
Comparison between experimental and numerically predicted by the FE model and the CZM stripping patterns for different values of $G_{IC}$.

The first step is to calibrate the CZM’s traction-separation law in terms of the experimental stripping pattern. Since the stripping is mode-I dominated, the $G_{IC}$ needs to be calibrated. In Figure 7, the experimental stripping pattern is compared with stripping patterns predicted using different values of $G_{IC}$. With blue color, the elements of the cohesive zone are presented, while with the brown color the deleted elements (stripped area) are presented. As shown, for $G_{IC}=1.02\;mJ/{\mathrm{mm}}^{2}$ no stripping is predicted by the model, while for values between 0.95 and 0.99 mJ/mm^2^ a stripping of the annular shape appears. With increasing the $G_{IC}$, the thickness of the ring increases, thus approaching the experimental stripping pattern. The best agreement between the test and the model is achieved for $G_{IC}=0.95\;mJ/{\mathrm{mm}}^{2}$, which is the selected value.

### 7.2. Analytical Stress Analysis

**Figure 8:**
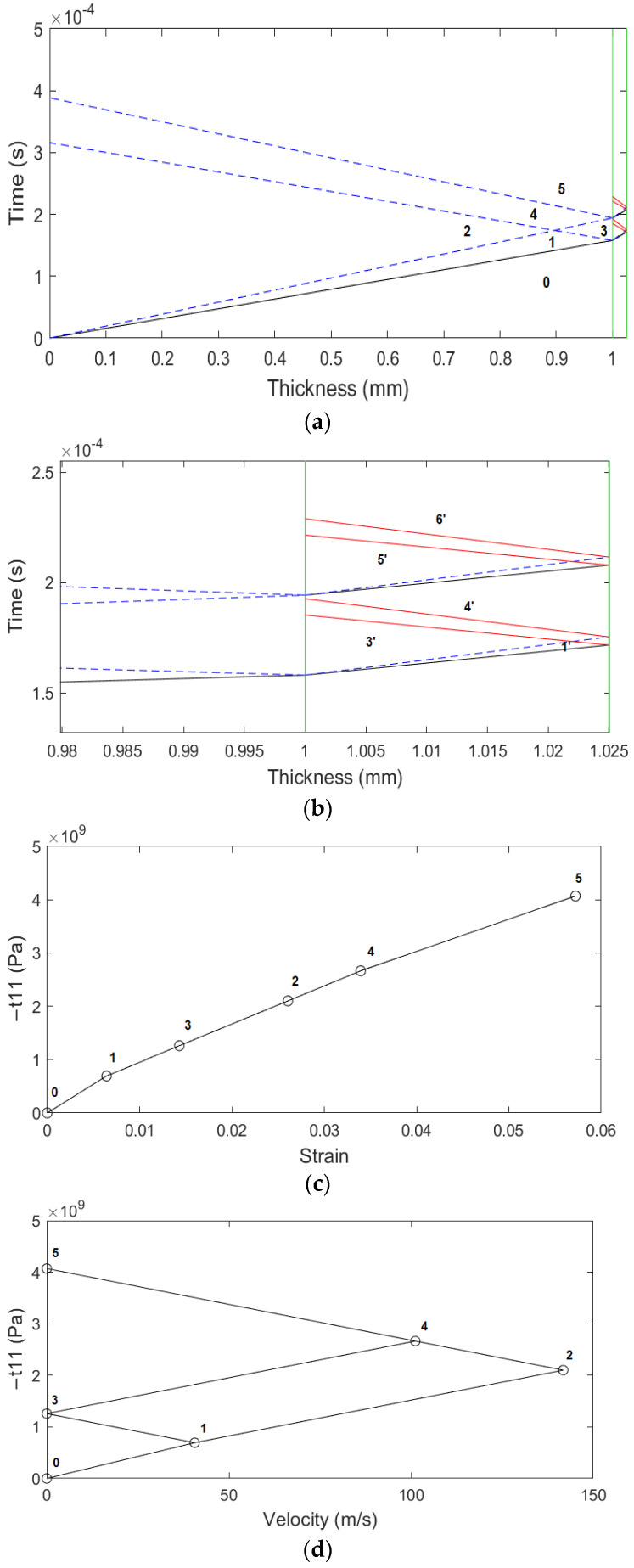
(**a**) Shock wave propagation in aluminum and epoxy material, (**b**) the shock wave interacts with the interface between the aluminum and the epoxy, (**c**) the stress–strain Hugoniot curve, (**d**) the stress–material velocity Hugoniot curve.

**Figure 9:**
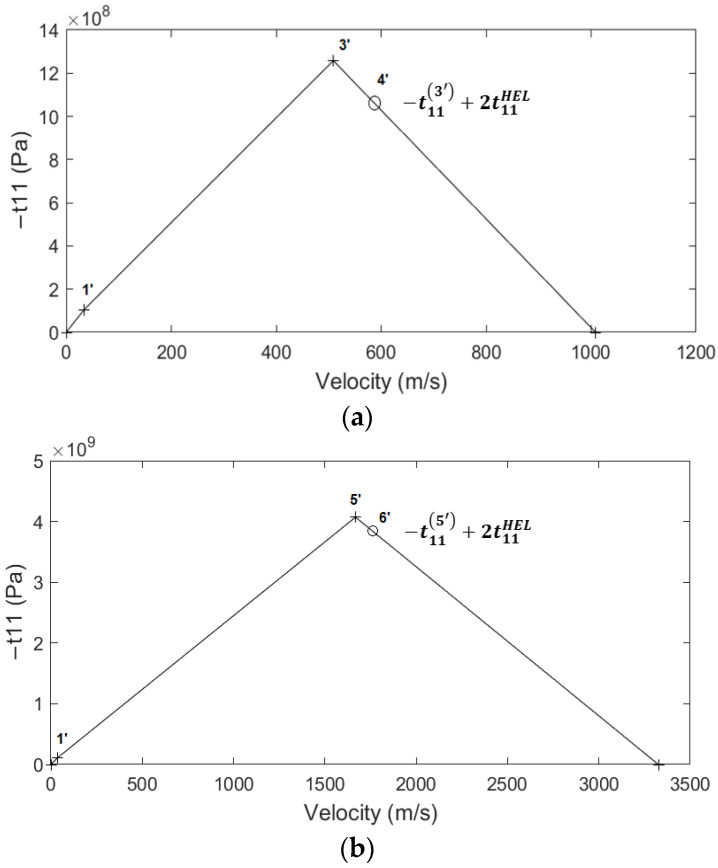
The stress–velocity Hugoniot curve for the (**a**) first and (**b**) second release wave inside epoxy.

Figure 8a plots the different states created by the shock wave inside the aluminum (thickness: 0–1.0 mm), while Figure 8b shows a closer view of the shock wave propagation inside the epoxy material (thickness: 1.0–1.025 mm). Figure 8c,d plots the stress–strain and stress–velocity Hugoniots for the aluminum material, respectively. Because the interface does not accelerate [48], the stress at state 3 will be the new boundary condition of stress for the study of the shock wave that will propagate into the epoxy material. This means that the applied stress to the epoxy is at the high-stress amplitude range [18]. A simple Hugoniot curve for the epoxy material is shown in Figure 9a,b. The stress of the elastic-plastic decompression shock that takes the material from state 3′ to state 4′ and from state 5′ to state 6′ is $2t_{11}^{HEL}=210\;\mathrm{MPa}$ and it is of great importance because it can be compared with the interface’s tensile strength or the spall fracture strength. It is noteworthy that the above applies only when the stresses are located in the high-stress amplitude range [18] and depends only on the mechanical properties of the material at the back free surface [22].

#### 7.2.1. Analytical vs. Numerical Stress Analysis

**Figure 10:**
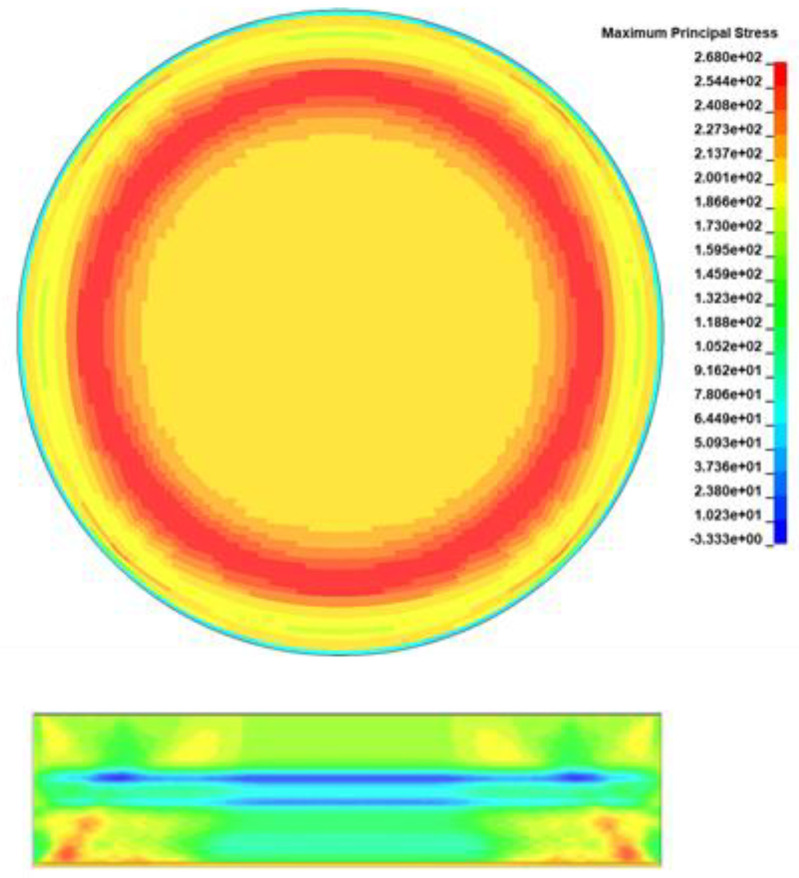
A representative numerical stress field.

In this section, the tensile stresses computed by the analytical model are compared with the numerical stresses. In Section 5, we showed that the parameters affecting the *HEL* values and consequently the magnitude of the tensile stress is the yield strength and the Poisson’s ratio of the epoxy. Since the exact values of these properties are not available, for the sake of the comparison, a parametric study has been conducted. In the study, the yield strength varied from 40 to 80 MPa and the Poisson’s ratio from 0.30 to 0.35. As shown in Figure 10, the numerical stress field is annular, which is explained by the corona effect [11]. Due to the irregular reflection of the shock wave front [49,50,51], a Mach stem is created close to the back free surface. The compressive stress field remains at the center while tensile stresses take place around it, forming a tensile stress ring. Therefore, for the comparison with the analytical model, which provides a single stress value as it is 1D, both the average and the maximum numerical stresses will be used.

**Figure 11:**
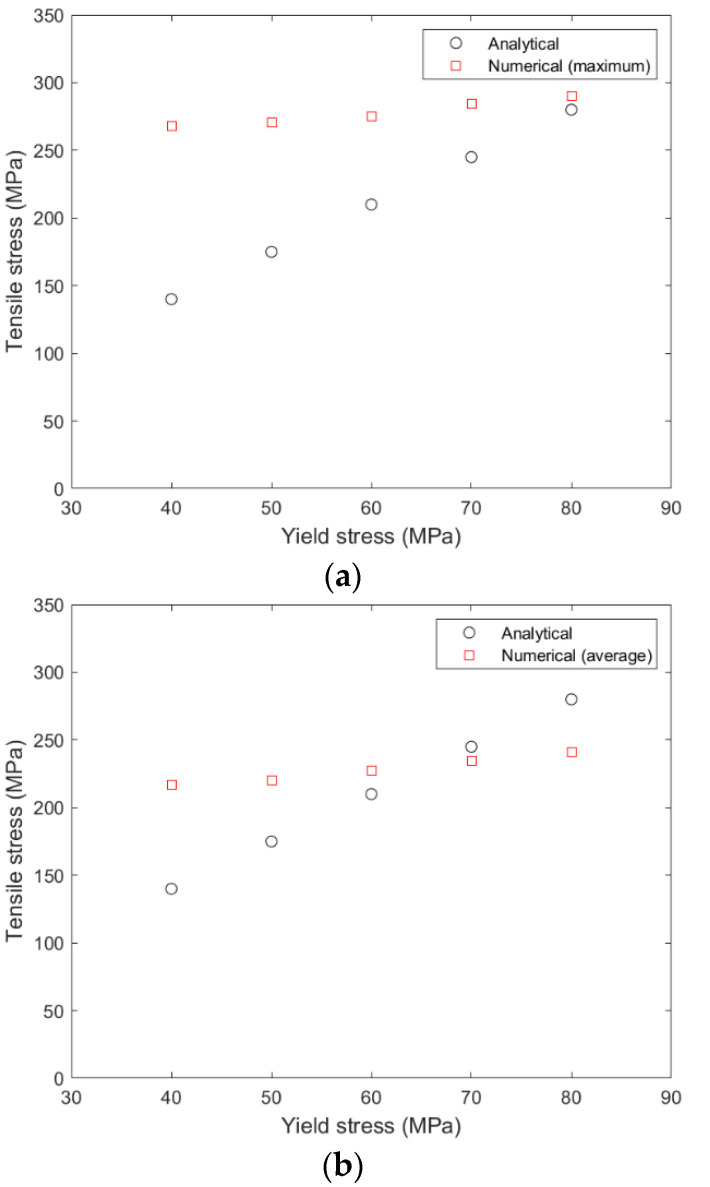
(**a**) Variation of the analytical and the maximum numerical stress in the epoxy with regards to the yield stress of the epoxy, (**b**) variation of the analytical and the average numerical stress in the epoxy with regards to the yield stress of the epoxy. The Poisson’s ratio is 0.3.

**Figure 12:**
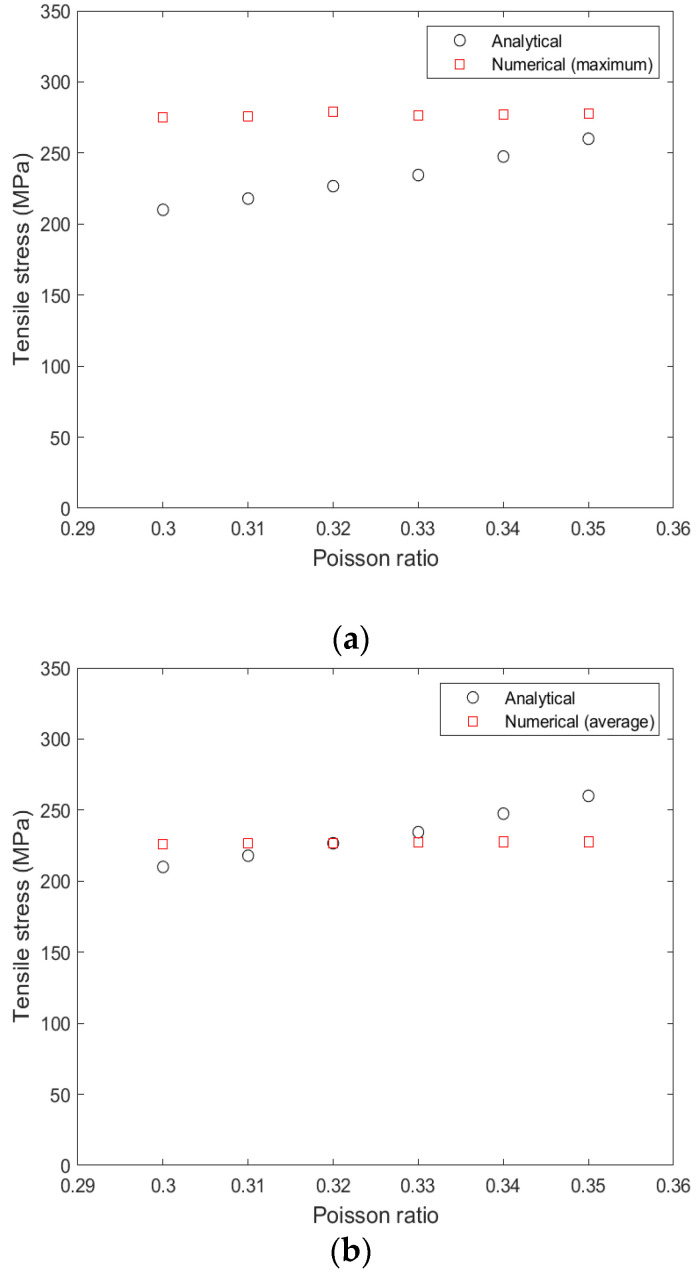
(**a**) Variation of the analytical and the maximum numerical stress in the epoxy with regards to the Poisson’s ratio of the epoxy, (**b**) variation of the analytical and the average numerical stress in the epoxy with regards to the yield stress of the epoxy. The yield stress is 40 MPa.

Figure 11 and Figure 12 compare the variation of analytical and numerical (maximum and average) tensile stresses in the epoxy with regards to the yield stress and the Poisson’s ratio of the epoxy, respectively. It is shown that the yield stress of the epoxy has a greater influence on the tensile stress than the Poisson’s ratio. That influence is greater for the analytical model than for the numerical model. For low values of the yield stress (40–70 MPa), there is a large deviation between the two models, which is larger between the analytical stress and the maximum numerical stress (Figure 11a), while for values of yield stress higher than 70 MPa, there is a better correlation. As shown in Figure 12, the influence of Poisson’s ratio is zero for the numerical model and minor for the analytical model, in comparison with the reference value of 0.3. For a yield stress of 60 MPa and Poisson’s ratio of 0.32 and 0.33, the analytical stress coincides with average numerical stress (Figure 12b).

#### 7.2.2. Enhancement of Interfacial Stress

**Figure 13:**
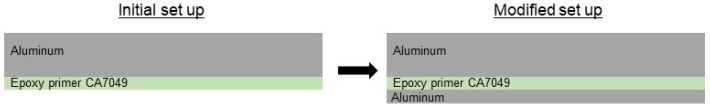
Difference in material set up after the addition of aluminum tape in the FE model.

In this section, we present a method to amplify the tensile stress at the aluminum/epoxy interface by exploiting the analytical and numerical results of the previous sections. As previously mentioned, the maximum tensile stress at the epoxy/aluminum interface is limited by the quantity $2t_{11}^{HEL}$, which is a function of the yield stress and the Poisson’s ratio. The aluminum has greater yield stress and Poisson’s ratio in comparison to the epoxy material. This generates the idea to place an aluminum film at the back free surface of the specimen, below the epoxy material as shown in Figure 13, so as to increase the tensile stresses due to its larger $2t_{11}^{HEL}$. With this configuration, the release wave that will be developed inside the aluminum is stronger than this inside epoxy material.

**Figure 14:**
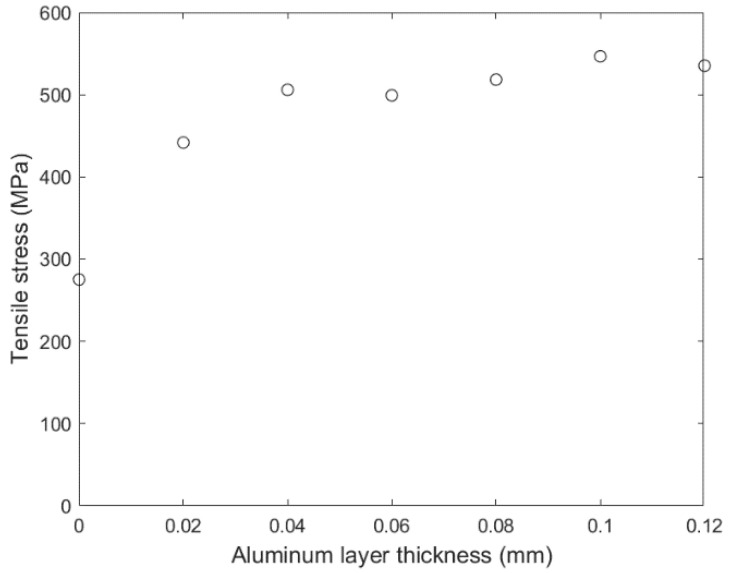
Variation of maximum tensile stress as predicted by the numerical model with the thickness of the added aluminum layer.

A short parametric study was performed, using the FE model, by varying the thickness of the added aluminum layer. The results are shown in the diagram in Figure 14. As shown, with increasing the thickness of the aluminum layer, the maximum tensile stress at the interface increases. The increase in the maximum tensile stress reaches a plateau at 0.08 mm. The maximum increase is from 277 MPa to 546 MPa, which is quite impressive.

### 7.3. Spall Fracture Prediction

In this section, an approximation of the stripping stress threshold in the form of spall fracture of the aluminum/epoxy interface is presented. The methodology requires as input the fracture properties of the aluminum/epoxy interface; however, these properties could not be measured experimentally due to the difficulty in the manufacturing of the respective specimens. The authors have made many trials to manufacture double cantilever beam and end-notch flexure specimens but the curing of the thin epoxy film between the aluminum adherents was not feasible. Consequently, we have drawn the properties from the literature [32,47,48].

The accuracy of Equation (25), which is crucial for the present implementation, was evaluated through the comparison with experimental results for the 90W–Ni–Fe alloy taken from [52]. Using the mechanical properties from [52] and the fracture toughness value of 60 MPaˑm^1/2^ from [53], the calculated and experimental spall strength of the 90W–Ni–Fe alloy is in full agreement (6.46 GPa). This result was achieved for a strain rate of 10^6^ s^−1^.

The stress threshold was calculated using Equation (28). The interface’s elastic modulus was derived using the following expression

(30) $$E=\frac{K^{2}}{G_{c}}$$

where K is the interface’s fracture toughness and 1$G_{C}$ is the critical fracture surface energy. K was taken equal to 23.71 Nˑmm^−3/2^, which is the average experimental value of [47]. $G_{C}$ was calculated equal to 125.27 J/m^2^ by Equation (29) using the average experimental value of [47]. E was used to calculate the shock wave *c* speed by $c=\sqrt{E/\rho }$ for various densities ρ of the interface between 900–1100 kg/m^3^. Knowing the shock wave speed at the interface and using the range of its thickness from 1 to 100 nm [32], the time domain t was then derived and used in Equation (28) to calculate the stress threshold. γ was taken equal to 232 mJ/m^2^ from [54]. Some further evaluation for interface thickness from 100 to 500 nm was made.

**Figure 15:**
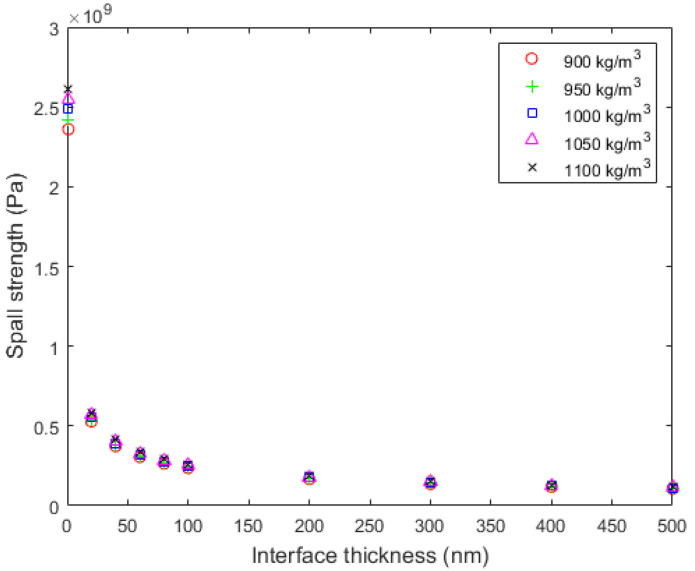
Variation of spall strength with the thickness of the aluminum/epoxy interface.

Figure 15 plots the spall fracture strength for different values of the interface thickness. For a small interface thickness of 1 nm, the threshold is in the range of 2.4–2.6 GPa. As the thickness increases to 20 nm, the threshold goes down to 580 MPa and for a thicker thickness of 100 nm and 500 nm, it is 260 and 110 MPa, respectively. Thus, it is safe to say that thickness plays a critical role in spall strength. On the contrary, the interface density slightly affects the spall strength.

Using Equations (25) and (26), the parameters of Table 1, and the fracture toughness properties from [55] the spall strength of the aluminum was also computed as follows:

$$P_{s}^{B}=3.58\;\mathrm{GPa},\;P_{s}^{B^{d}}=5.68\;\mathrm{GPa},\;P_{s}^{D}=2.78\;\mathrm{GPa}$$

where $P_{s}^{B}$ is the brittle spall strength computed using the quasi-static fracture toughness from [55], $P_{s}^{B^{d}}$ is the brittle spall strength computed using the dynamic fracture toughness from [55], and $P_{s}^{D}$ is the ductile spall strength. All values are higher than the maximum tensile stresses computed by the analytical and numerical models and thus no spall initiation is expected in the aluminum.

#### 7.3.1. Numerical Simulation of Stripping Using the Spall Strength

**Figure 16:**
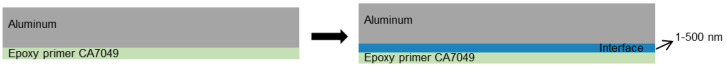
Difference of material set up after the addition of interface.

Using the spall strength values in a continuum progressive damage model, stripping initiation and propagation can be simulated as an alternative tool to the CZM model. The interface was modeled by solid elements using the same material model and properties as the epoxy, as shown in Figure 16, except density, which was taken as the average value of Figure 15 equal to 1000 kg/m^3^. The interface elements were eroded using an eroding parameter in MAT_000_ADD_EROSION, which is activated when the maximum principal stress of the element reaches the spall strength. Analyses have been performed for different combinations of the interface thickness and spall strength, taken from Figure 15.

**Figure 17:**
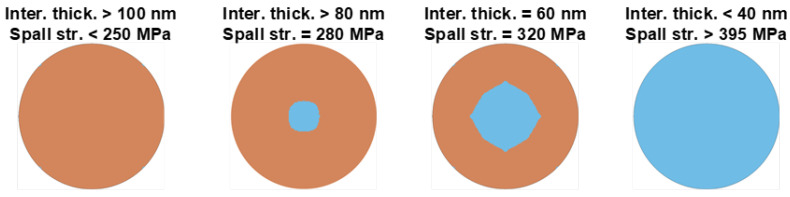
Stripping patterns predicted by the combination of the FE model and the spall strength for different interface thicknesses.

The results are illustrated in Figure 17. For interface thickness larger than 100 nm and spall strength lower than 250 MPa, the model predicts full stripping. For lower values of interface thickness (higher values of spall strength), an annular stripping pattern is predicted. The thickness of the stripping ring decreases with decreasing the interface thickness. The differences in the stripping patterns predicted by the CZM (Figure 7) lie in the fact that the CZM erodes elements using a displacement threshold and the continuum damage using a stress threshold.

#### 7.3.2. Analytical Prediction of Stripping Using the Spall Strength

As a final approach, the analytical model and the spall strength model are combined to obtain a very fast prediction of stripping initiation. To this end, the analytical stress at the epoxy is compared with the spall strength of the interface based on the assumption that stress at the epoxy is close to the stress at the interface [48]. The maximum tensile stress computed by the analytical model (210 MPa) is greater than the minimum spall strength (110 MPa) and thus, stripping is predicted using the two analytical models. Given that the numerical model predicts stripping using the spall strength of 110–320 MPa (Figure 17), it becomes evident that the analytical model underestimates the magnitude of the tensile stress field developed at the epoxy and the interface.

## 8. Discussion

The FE model with the CZM is the most accurate tool, although in most cases it is the most computationally expensive. Its only drawback is the lack of experimental fracture toughness properties for the aluminum/epoxy interface. However, after the calibration of those properties, conducted against the experimental stripping pattern, it becomes a very useful tool for computing the stress field at the aluminum/epoxy interface and for simulating stripping. The specific model has been successfully used in [12] to conduct a parametric study on the effect of laser and material parameters on the stripping pattern and can be used in the future for the virtual testing and optimization of the laser shock paint stripping process.

The spall fracture model has given trusted estimations of the spall strength of the interface which are very sensitive to the interface thickness and less sensitive to the interface density. The spall strength values when incorporated into a continuum damage model can predict the stripping initiation. However, such a model requires more data for the interface such as the thickness and Young’s modulus. If these data are available, the spall-based continuum damage model might be used as an alternative to the CZM model. In addition, the spall fracture model can be used for checking for the non-desired spall fracture of the aluminum substrate.

**Figure 18:**
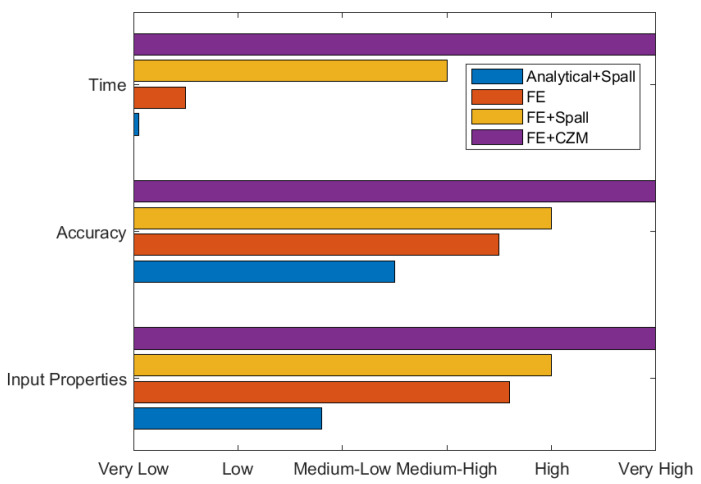
Qualitative comparison of the different models that predict spallation of the interface.

The analytical stress analysis model can be used to efficiently represent the shock wave propagation into the material system, but it can give only a preliminary estimation of the tensile stress at the epoxy, which is very sensitive to the yield stress and Poisson’s ratio of the epoxy. As it is 1D, it cannot capture the stress variation while the estimated tensile stress is closer to the average numerical stress rather than the maximum stress. By assuming that the epoxy stress is close to the interface stress, the analytical stress can be compared to the spall strength to predict stripping. In the present application, this prediction has been proven not efficient enough. Figure 18 shows a qualitative comparison of the different approaches in terms of required time, accuracy, and input properties.

## 9. Conclusions

**Figure 19:**
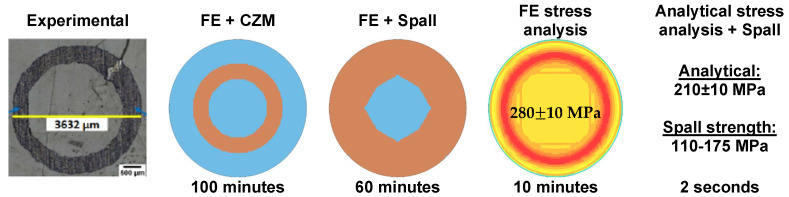
Stripping patterns predicted by the combination of the FE model and the spall strength for different.

In the present paper, we have developed analytical and numerical models to compute the stress field and predict the failure of an aluminum/epoxy interface subjected to laser shock loading in the frame of the paint stripping technological problem. The developed models comprise a FE model combined with the CZM method, an analytical model for computing stresses due to shock wave propagation, and an analytical model for computing the spall strength of the aluminum/epoxy interface. The models have been combined to create computational tools for decreasing computational effort. Figure 19 schematically describes the main findings of the computational tools by also depicting the computational time. The computational time is expected to become a more important parameter in the modeling of the paint stripping on structural parts where repeated laser shocks will be applied.

## Figures and Tables

**Table 1:** Materials’ mechanical properties [11,12].

Parameter	Value
Aluminum	
Density	2700 kg/m^3^
Young’s Modulus	73 GPa
Poisson’s ratio	0.33
Yield strength	352 MPa
Strain hardening modulus	440 MPa
Strain hardening exponent	0.42
Strain rate coefficient	0.0083
Speed of the wave	5328 m/s
Linear Hugoniot slope coefficient	1.338
Gruneisen gamma	2
Epoxy	
Density	1700 kg/m^3^
Young’s Modulus	4.16 GPa
Poisson’s ratio	0.30–0.35
Yield strength	40–80 MPa
Speed of the wave	2000 m/s
Linear Hugoniot slope coefficient	1.493
Gruneisen gamma	1.13

## Data Availability

Not applicable.
